# On-chip polarization management for stable nonlinear signal generation in thin-film lithium niobate

**DOI:** 10.1515/nanoph-2025-0339

**Published:** 2025-11-10

**Authors:** Junhyung Lee, Sunghyun Moon, Yongchan Park, Uijoon Park, Hansol Kim, Changhyun Kim, Minho Choi, Jin-Il Lee, Hyeon Hwang, Min-Kyo Seo, Dae-Hwan Ahn, Hojoong Jung, Hyounghan Kwon

**Affiliations:** Center for Quantum Technology, 58975 Korea Institute of Science and Technology (KIST), Seoul 02792, South Korea; School of Electrical Engineering, Korea University, Seoul 02841, South Korea; School of Integrated Technology, Yonsei University, Seoul 21983, Republic of Korea; Department of Artificial Intelligence Semiconductor Engineering, Hanyang University, Seoul 04763, South Korea; Division of Nano & Information Technology, KIST School, Korea University of Science and Technology, Seoul 02792, Republic of Korea; Department of Physics, Korea Advanced Institute of Science and Technology (KAIST), Daejeon 34141, South Korea; Division of Quantum Information, KIST School, Korea University of Science and Technology, Seoul 02792, South Korea

**Keywords:** lithium niobate, periodically poled lithium niobate, second harmonic generation, feedback loop, on-chip polarization management, Stokes parameter

## Abstract

Nonlinear signal generation requires precise control of the input polarization to satisfy phase-matching conditions. Conventional polarization management using external fiber polarization controllers or bulk wave plates increases coupling complexity and can degrade polarization fidelity and conversion efficiency in nonlinear photonic systems. Here, we demonstrate on-chip polarization control in thin-film lithium niobate nonlinear photonic circuits. Integrated polarization modulators enable real-time tuning of arbitrary input polarization states and thus provide on-demand control of nonlinear conversion in a periodically poled lithium niobate waveguide. A closed-loop feedback system, which integrates auto-compensation and automatic fiber-chip alignment routines, automatically optimizes the second-harmonic generation intensity and maintains performance over extended periods despite polarization scrambling and environmental perturbations. This integrated approach reduces coupling complexity and offers a scalable route toward fully reconfigurable nonlinear photonic systems.

## 1 Introduction

Lithium niobate has emerged as a leading platform for second-order nonlinear optical interactions due to its wide bandgap, broad transparency window (0.35–5 μm), large second-order and ferroelectric coefficients [1], [2], [3], [4], [5], [6]. Nonlinear frequency conversion has been extensively investigated to access a wide range of wavelengths from the ultraviolet to the mid-infrared through second-order processes such as second harmonic generation (SHG), sum-frequency generation (SFG), and difference-frequency generation (DFG) [7], [8], [9], [10], [11], [12]. Achieving efficient frequency conversion requires strict phase matching, which ensures phase coherence among interacting waves and facilitates constructive interference over the propagation length [13]. To fulfill the stringent requirements of phase matching in nonlinear optical processes, several strategies have been devised, including birefringent phase matching, modal phase matching, and quasi-phase matching (QPM) [14], [15], [16], [17], [18]. QPM is an engineered phase matching technique that compensates the relative phase at regular intervals by means of ferroelectric domain inversion, leading to periodically poled lithium niobate (PPLN) [19], [20], [21]. Maximizing nonlinear conversion efficiency in PPLN requires precise control of the input polarization [3], [22]. Since QPM structures are typically designed to exploit type-0 phase matching, where all interacting waves are aligned along the extraordinary axis, the polarization state of the input must be carefully aligned with the crystal axis to ensure maximum overlap with the largest nonlinear tensor element *d*_33_ [6], [23].

Thin-film lithium niobate (TFLN) offers strong normalized nonlinear conversion efficiencies due to tight mode confinement in sub-micron waveguides and the possibility of engineering ferroelectric domain inversions with high precision [1], [3]. Intensive efforts have focused on PPLN waveguides for efficient frequency conversion processes which are fundamental to both classical and quantum photonics [18], [24], [25], [26], [27], [28]. Furthermore, the low propagation loss and large electro-optic (EO) coefficient of TFLN make it a leading contender for integration with modulators, resonators, and interferometers on a single chip, enabling multifunctional photonic circuits [5], [29], [30], [31], [32]. In particular, TFLN-based EO modulators have been successfully demonstrated in classical optical communication systems operating beyond 100 Gbit/s, as well as in quantum photonics platforms that require precise and high-speed control of optical phase and amplitude [31], [33]. However, such EO modulators are known to suffer from severe DC drift, a phenomenon in which the bias point gradually shifts over time [31], [34], [35], [36], [37], [38], thus compromising long-term stability. In contrast, thermo-optic (TO) modulation induced by resistive heaters on the waveguides provides robust and drift-free phase control. Although TO modulation is inherently slower than EO modulation, it can offer excellent stability, making it suitable for static tuning or long-term operation [34], [39].

Among diverse types of integrated optical modulators, on-chip polarization modulators consisting of polarization splitter rotators (PSRs) and beam splitters have been developed in conjunction with TFLN modulators to enable comprehensive manipulation of the polarization state [40]. These integrated devices support on-demand control of polarization. Despite the superior performance of the polarization modulators, they operate within the linear optical regimes and their applications for nonlinear devices have remained elusive [40], [41], [42], [43]. In addition, a recent active nonlinear photonic device composed of a Mach–Zehnder interferometer (MZI), a EO phase modulator, and a PPLN waveguide achieved modulation of nonlinear signal with high extinction ratio but still relied on off-chip polarization control [44].

Polarization management has traditionally relied on off-chip bulk components, such as fiber-based polarization controllers or external wave plates, which add complexity, introduce insertion loss, and hinder integration scalability [45]. While modern fiber polarization controllers can be compact and low-loss, the on-chip polarization state might remain uncertain. In addition, mechanical fiber controllers are relatively slow compared with thermo- or electro-optic on-chip actuators and piezoelectric fiber controllers, while faster, are generally more costly and power-consuming.

In contrast, on-chip polarization management permits precise control of the polarization and, when combined with an active feedback loop, can compensate for unwanted environmental changes such as temperature drifts or mechanical perturbations that ultimately alter the polarization state, as well as power-dependent polarization crosstalk. In this sense, the synergy of lithium niobate can be highlighted, where both active control and strong nonlinear effects are simultaneously harnessed to achieve robust polarization management for nonlinear photonic applications.

In the context of nonlinear optics, conventional fiber-based polarization controllers have long played an important role, but their function is limited to the input stage and does not directly stabilize the polarization inside the chip. Integrating polarization control with on-chip nonlinear signal generation overcomes these limitations and enables direct stabilization of the polarization [22], [24], [46].

In this work, the architecture includes balanced MZIs, PSRs, TO phase shifters and PPLN waveguides. We investigate the design and simulation analysis of fundamental building blocks, including the PSR and PPLN, for implementing an active polarization modulator. The modulator achieves full coverage of the Poincaré sphere by controlling two TO phase shifters, enabling arbitrary polarization control. Since SHG intensity scales with the square of the fundamental intensity projected onto the correct polarization, a polarization misalignment angle *θ* reduces the SHG output as *I*_SHG_ ∝ cos^4^ *θ*, so minor deviations lead to a marked decrease in output, highlighting the importance of arbitrary polarization control. By using an active feedback control loop to optimize the SHG intensity, we demonstrate that the SHG output can be maximized regardless of the input polarization state [31]. Moreover, the adoption of auto-compensation and automatic fiber-chip alignment systems allows the optimized output to be maintained despite polarization scrambling and environmental vibrations. These findings demonstrate the feasibility of fully integrated on-chip polarization control for stable nonlinear signal generation in TFLN, offering practical advantages for reconfigurable and high-performance integrated photonic circuits.

## 2 Results

### 2.1 Conceptual principles of a frequency converter

**Figure 1:**
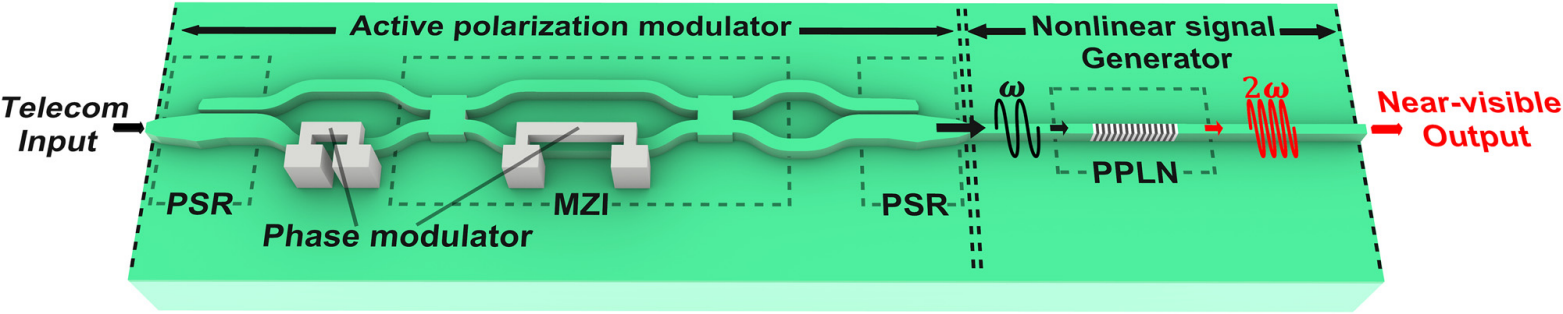
Conceptual schematic of the PPLN-based frequency converter. The active polarization modulator consists of two PSRs, phase modulators and an MZI. The nonlinear signal generator includes type-0 PPLN waveguide. Active polarization control precisely adjusts the polarization state, enabling continuous tuning of the nonlinear signal intensity for efficient telecom-to-visible wavelength conversion.

Figure 1 shows the conceptual schematic of the PPLN-based frequency converter, which is divided into two regions, comprising an active polarization modulator and a nonlinear signal generator. In the region of the active polarization modulator, the incoming telecom-band signal passes through two PSRs, an MZI, and two TO phase shifters. By applying precisely tuned voltages to the TO phase shifters, the input polarization state is adjusted to either satisfy or even intentionally deviate from the type-0 phase-matching condition in the subsequent X-cut PPLN waveguide [40]. This waveguide supports only the fundamental TE mode for efficient conversion. In the region of the nonlinear signal generator, the PPLN waveguide with periodically poled domains to tailor the nonlinear coefficient, facilitates frequency up-conversion from telecom to near-visible light. The precise polarization alignment can adjust the spatial overlap between the guided optical modes and the nonlinear coefficient distribution, thereby ensuring efficient modulation of the up-conversion [47]. Hence, active polarization control of the fundamental TE mode enables optimized type-0 phase matching. The architecture depicted in Figure 1 enables arbitrary control of the TE and TM polarization ratio at the input of the PPLN region, independent of the incident polarization state. This capability allows active management of the nonlinear signal.

In the PSR section of the active polarization modulator, light is routed through two closely spaced TFLN waveguides where the optical path is varied adiabatically according to the input polarization. A TE_0_ mode stays confined to the lower waveguide, while a TM_0_ input rotates into the TE_1_ eigenmode and is then transferred laterally to the upper waveguide, where it becomes the TE_0_ eigenmode. Measured extinction ratios are on the order of 10–20 dB, and this controlled mode conversion depends on carefully engineered intermodal coupling [48] (see Supporting Note 1 and Figure S1 for details).

In the PPLN section of the nonlinear signal generator, by inverting the sign of the second-order nonlinear coefficient in periodic manner, the PPLN structure introduces an effective grating wavevector that compensates the intrinsic phase mismatch and generates nonlinear signal [21]. The corresponding poling period for QPM was computed for each waveguide width by using the effective refractive indices at the pump and signal wavelengths. These results demonstrate how QPM allows precise tailoring of phase matching through geometric and material design parameters (see Supporting Note 2 and Figure S2 for details).

The TFLN wafer (supplied by NanoLN) including an X-cut 500 nm thick top TFLN layer, buried a 4.7 μm thick SiO_2_, and a 525 μm thick Si substrate is prepared. The fabrication procedures of the active polarization modulator are described in detail (see the methods and Supporting Note 3).

### 2.2 Arbitrary polarization modulation

**Figure 2:**
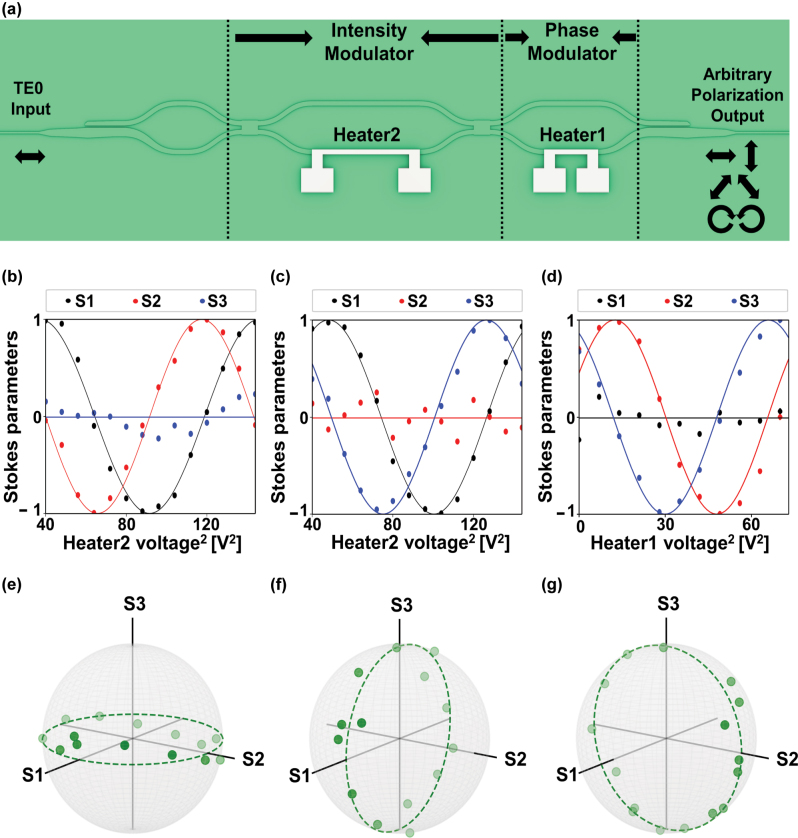
On-chip arbitrary polarization modulation. (a) Schematic of the polarization modulation scheme employing an intensity modulator (IM) and a phase modulator (PM). (b) and (c) Measured Stokes parameters S_1_ (black), S_2_ (red) and S_3_ (blue) as functions of heater2 voltage, with heater1 biased to introduce a *π*/2 phase offset between the two traces. (d) Measured Stokes parameters S_1_ (black), S_2_ (red) and S_3_ (blue) as functions of heater1 voltage for a fixed phase bias on heater2. In all panels, solid curves are sinusoidal fits to the data. The corresponding sample points for each case are individually extracted and mapped on the Poincaré sphere: (e) S_1_–S_2_ plane, (f) S_1_–S_3_ plane, and (g) S_2_–S_3_ plane.

In this section, we experimentally demonstrate that the proposed modulators enable arbitrary control of the TE and TM polarization ratio at the input of the PPLN region, regardless of the input polarization state [40]. To verify such capability, we exploit time-reversal symmetry and characterize the polarization transformations by alternating the direction of light propagation. Specifically, we launch TE-polarized telecom light into the waveguide containing the PPLN region, apply electrical signals to the heaters, and analyze the resulting output polarization states. Our measurements reveal significant coverage of the Poincaré sphere, confirming arbitrary polarization manipulation. The measurement was carried out in free space using wave plates and a polarizer, as detailed in the Section 4.3, subsection of the Methods section. As depicted in Figure 2(a), a balanced MZI first redistributes the input TE_0_ power between its two arms, enabling precise generation of the horizontal, vertical, diagonal, and anti-diagonal polarization states by controlling the applied voltages on heater2. The heater1 is required to generate a relative phase shift between paths, which is employed to give the exact phase offset required for the elliptical polarization states (see Supporting Notes 4, 5 and Figure S4(b) for details).

Crucially, by independently sweeping both heaters over their full tuning ranges, we recorded complete polarization trajectories on all three Stokes parameter planes (S_1_−S_2_, S_1_−S_3_ and S_2_−S_3_), as shown in Figure 2(b)–(d). In each projection, two Stokes components exhibit clear sinusoidal lines with the expected *π*/2 phase delay relationship while the third remains fixed at zero. Stokes parameters S_2_ and S_3_ both depend on the voltages applied to heater1 and heater2. Changing only one heater’s voltage produces a sinusoidal variation in each parameter, and the overall intensity of S_2_ or S_3_ equals the product of those two sinusoids. As a result, the shapes of the S_2_ and S_3_ curves versus heater2 voltage vary with the heater1 bias. In Figure 2(b) heater1 is set so that S_2_ reaches its peak exactly where S_3_ maintains zero. In Figure 2(c) heater1 is adjusted so that S_2_ maintains zero exactly where S_3_ reaches its maximum. Figure 2(d) presents the Stokes parameter modulation driven by heater1 while heater2 is held at a fixed phase bias. In Figure 2(b)–(d), the solid lines represent the sinusoidal and linear fits to S_1_, S_2_ and S_3_ and the deviations between the measured data and these fits arise from the discrete voltage steps applied to the heaters. That is mainly because the applied voltages are swept in a discrete manner and the step sizes are non-negligible. All of the corresponding paths on the Poincaré sphere, as shown in Figure 2(e)–(g), trace continuous great circles, extending from the equatorial plane up to the north and south poles. These high fidelity rotations demonstrate that our device achieves significant coverage of the Poincaré sphere across all polarization bases when the input light is TE_0_-polarized. Furthermore, a detailed theoretical analysis based on Mueller matrices is provided in Supporting Notes 4, 5 and Figures S4(b), S5.

### 2.3 Tunable nonlinear signal output via polarization control

**Figure 3:**
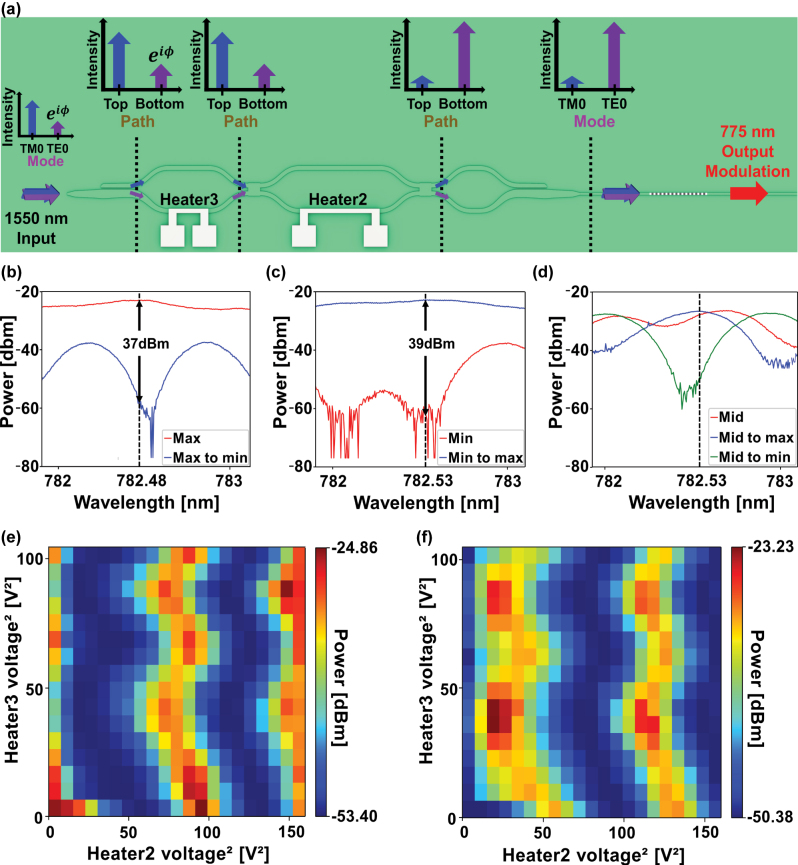
Tunable nonlinear signal output via polarization control. (a) Schematic of the nonlinear signal intensity controller. The TE_0_ mode for type-0 phase matching is precisely controlled with two PSRs, phase modulators and an MZI. (b) Measured extinction ratio between the maximum SHG intensity and the modulated SHG intensity down to minimum level. (c) Measured extinction ratio between the minimum SHG intensity and the modulated SHG intensity up to maximum level. (d) Active modulation of an intermediate SHG intensity that can be driven both up to the maximum and down to the minimum. Magnitude distribution of SHG signal as a function of applied voltages on heater for two initial conditions with both heaters unbiased: (e) maximum SHG intensity and (f) minimum SHG intensity.

For tunable nonlinear signal output via polarization control, the input direction of the incident telecom light is reversed relative to the input configuration used in the previous section. Our device is capable of mapping an arbitrary input polarization back into the TE_0_ mode, as shown in Figure 3(a). The input PSR converts the polarization state into a path-encoded signal, while the output PSR maps the path information back into polarization. The central MZI, equipped with two thermo-optic phase shifters, modulates the power splitting ratio between the two arms, thereby controlling the relative amplitudes of the TE_0_ and TM_0_ modes at the PPLN input. Specifically, when the input polarization is aligned to a circular state with a phase difference *ϕ* between the TE_0_ and TM_0_ modes, the symmetric MZI could route all optical power into a single arm, limiting modulation flexibility [49]. To avoid this, we apply an offset to compensate the phase difference *ϕ* between the TE_0_ and TM_0_ modes using heater3, ensuring that heater2 supports the desired tunable operation. This configuration provides robust, continuous control over the TE_0_/TM_0_ balance, and thus enables fine-tuning of the SHG intensity at 775 nm under 1,550 nm excitation (see Supporting Note 4 and Figure S4(a) for details).

Figure 3(b) and (c) present two complementary SHG modulation results enabled by the dual-heater configuration. As shown in Figure 3(b), the SHG output is attenuated from its maximum (TE_0_) to minimum (TM_0_) level, while the SHG signal is enhanced from minimum to maximum level, as shown in Figure 3(c). Initial polarization states were established using an off-chip polarization controller and then switched between the TE_0_ and TM_0_ modes by adjusting the heater voltages and the dotted lines in Figure 3(b)–(d) indicate the wavelengths at which the intensity is maximal when the polarization state reaches its peak. The measured extinction ratios reach up to 39 dB, which are comparable to those obtained with the off-chip polariation controller, and all measurements approach the detector sensitivity limit (a few nanowatts) (see Supporting Note 6 and Figure S6(e) for details). Together, these results highlight the excellent active modulation range of the nonlinear system. Figure 3(d) demonstrates that any intermediate SHG level can be precisely controlled with the heaters2 and 3, enabling deterministic modulation toward either the high or low transmission state. We should mention that while active nonlinear modulations with high extinction ratios have been demonstrated in TFLN platforms, such devices still rely on the off-chip polarization controller for the desired nonlinear effects [44].

Figure 3(e) and (f) illustrate the SHG intensity as a function of the voltages applied to heater2 and heater3 for two representative input polarization states. Figure 3(e) shows the measured nonlinear signal as a function of the two heater voltages when the input polarization state is aligned near the optimal phase-matching condition. Similarly, Figure 3(f) presents the corresponding results when the input polarization state yields minimal SHG efficiency. These measurements validate that the dual-heater architecture enables continuous and precise control of the SHG output across a broad active range for two orthogonal input polarization states.

### 2.4 Closed-loop active control of nonlinear signal intensity

For integrated nonlinear photonic devices, where fiber interfaces are commonly used to couple light into the devices, polarization degradations can be further exacerbated by environmental changes or mechanical deformations of the fiber. These unwanted fluctuations in the polarization can consequently lead to instability in the nonlinear interaction. In nonlinear optics, small misalignments of the input polarization from the optimal orientation can cause considerable reductions in SHG intensity, which approximately follow a cos^4^ dependence on the misalignment angle. To address these challenges, we integrate a closed-loop feedback controller with our polarization-modulated SHG modules. This system continuously samples the SHG output via a photodetector and feeds the signal to a control computer, which adjusts the heater voltages to steer the polarization state toward the desired setpoint. This real-time feedback scheme is expected to effectively counteract thermal drift, mechanical vibrations, and other environmental disturbances.

**Figure 4:**
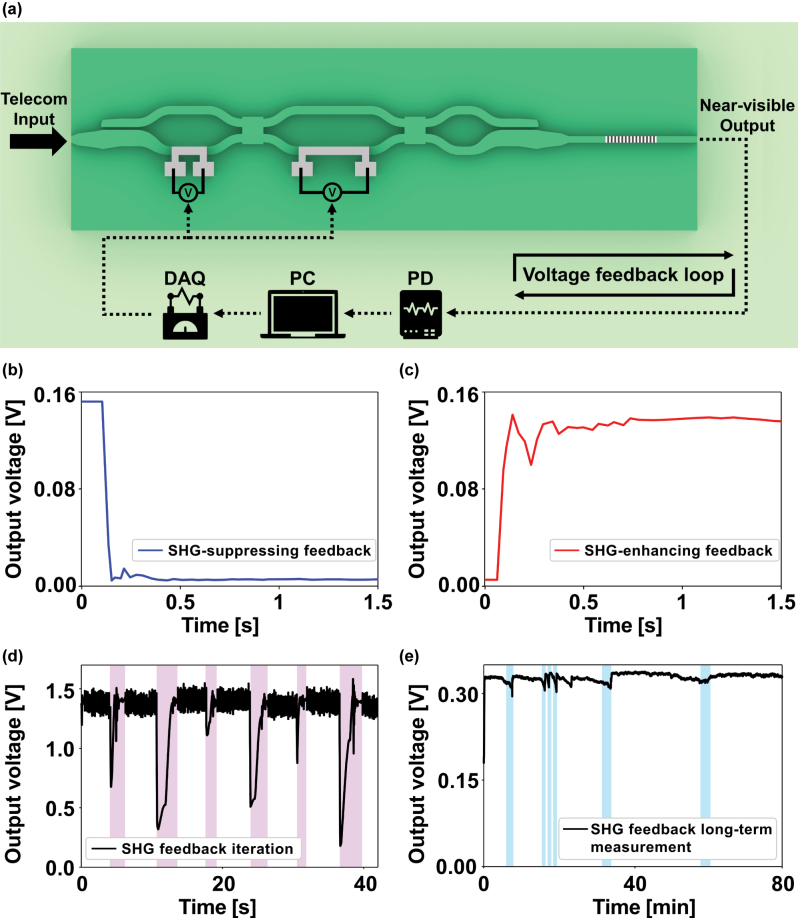
On-chip closed-loop active control of nonlinear signal intensity. (a) Schematic of the automated feedback loop. (b) SHG signal suppressing feedback (from TE_0_ to TM_0_). (c) SHG signal enhancing feedback (from TM_0_ to TE_0_). (d) SHG signal feedback iteration. Shaded pink region denotes periods of re-optimization. (e) Long-term SHG stability of the feedback loop. Shaded blue region denotes periods of re-alignment.

Figure 4(a) shows the feedback loop implemented in our device. At each step, we record the SHG intensity on a photodetector immediately before and after a small heater-voltage adjustment, then compare the two readings to decide the next voltage increment. To steer the system toward either a maximum or a minimum of the SHG response, we employ the Nelder–Mead simplex algorithm. Because the SHG intensity varies sinusoidally with heater voltage, the sign of the second derivative at the starting point determines whether the simplex converges to a peak or a trough. In regions where the curvature is negative (concave down), it is drawn toward a maximum, whereas positive curvature (concave up) drives it to a minimum.

To resolve this ambiguity at the outset, we first locate the nearest extremum relative to our reference phase-matching point *P*_*π*_, a half-period phase shift in the SHG signal. We do this by sampling several points in the vicinity of *P*_*π*_ and identifying which side of the inflection the signal lies on, thereby determining whether the closest extremum is a maximum or a minimum. Once the correct extremum is identified, we initialize the Nelder–Mead search within that local region, ensuring robust convergence to the intended target (see Supporting Note 7 and Figure S7 for details). Figure 4(b) and (c) show the results of these routines, illustrating SHG intensity suppression and enhancement, respectively. Modulating the polarization between TE_0_ and TM_0_ suppresses or enhances the SHG signal. By continuously recording each measurement and using it to inform the subsequent voltage update, the device converges to the desired nonlinear signal level in approximately 0.5 s despite external perturbations.

To evaluate robustness, we measured the feedback system’s response to repeated polarization disturbances and its long-term behavior. Figure 4(d) demonstrates that the SHG intensity can be restored following severe polarization degradation. The shaded pink area in Figure 4(d) indicates periods of random polarization fluctuations experimentally induced by random rotations of a conventional mechanical polarization controller. The automated feedback system continuously adjusts the heater voltages and recovers the SHG output. Figure 4(e) shows that the output intensity and the SHG power can be stable over an hour. The SHG intensity typically decreases after a few minutes due to slight fiber-chip misalignment caused by environmental fluctuations. To mitigate this, we implemented an auto fiber-chip alignment routine that triggers re-alignment whenever the intensity falls below a preset fraction of the initial value and the shaded blue region in Figure 4(e) marks this fiber-chip re-alignment process. Using this procedure, the feedback loop restores and maintains the maximized SHG output for over 1 h of continuous operation. Overall, the results demonstrate that the proposed structure exhibits high long-term stability and robustness under severe polarization-degrading conditions.

## 3 Discussions

In this work, we have presented on-chip polarization management for stable nonlinear signal generation in TFLN. Our results demonstrate that the polarization modulator, composed of PSRs and MZI-based phase shifters, achieves precise polarization alignment for type-0 phase matching, leading to highly efficient SHG. Compared to prior implementations, on-chip arbitrary polarization control has been demonstrated previously, primarily in the telecom band using EO modulators with fast switching [40]. In contrast, we aim to demonstrate long-term polarization stability on a nonlinear photonic platform by employing TO tuning and the tuning bandwidth can be extended into the kilohertz range [34]. Our devices have PSRs with relatively low extinction ratio and MZIs with high extinction ratio, and operate with driving voltages below 10 V.

Intermodal adiabatic coupling in our PSR design already enables robust mode conversion across the entire telecom C-band, with simulated extinction ratios of 30 dB for TE_0_ and 20 dB for TM_0_ at 1,550 nm. However, improving these extinction ratios will directly boost device performance by reducing residual crosstalk, sharpening spectral selectivity, and increasing conversion efficiency in quantum frequency conversion and classical communication systems. Future PSR designs that fully harness the broad range of the SHG response through optimized waveguide geometry and precise phase matching will deliver higher contrast and enable greater signal fidelity as well as lower noise for advanced nonlinear optical applications.

In our discussion of the dual-heater architecture for arbitrary polarization generation (Figure 3) and active SHG intensity control (Figure 4), we emphasize both full functionality and minimal-component operation. In the full configuration using heaters1, 2, and 3, any input polarization states including arbitrary elliptical polarization can be mapped to any polarization state, thereby fully spanning the Poincaré sphere and providing complete on-chip control over all Stokes parameters (see Supporting Note 4 and Figure S4(c) for details). However, if the goal is only to convert a linear input polarization to desired elliptical state, a reduced arrangement of heater2 followed by heater1 is sufficient. Conversely, to recover a pure linear state from an arbitrary elliptical input, only heater3 upstream of heater2 needs to be engaged. Thus, by selecting heater combinations appropriately, such as heaters1 and 2 for linear to arbitrary conversion, heaters2 and 3 for arbitrary to linear mapping, or all three heaters1, 2 and 3 for arbitrary to arbitrary transformation, the platform delivers both streamlined implementations and full versatility for advanced nonlinear signal engineering on chip.

However, during polarization control using heaters, the crosstalk that one heater influences the behavior of another can occur. In cases where a single heater is expected to affect only a specific Stokes parameter, we observe that the other heater can also alter the parameter’s intensity. This crosstalk introduces unintended phase shifts and polarization drift, thereby limiting independent tuning of the Stokes parameters and reducing the predictability of SHG optimization. By characterizing this behavior, nevertheless, we developed a fitting approach that reproduces the measured Stokes parameters with high accuracy (see Supporting Note 5 and Figure S5 for details).

As shown in Figure 4(b) and (c), the Nelder–Mead–based feedback loop converges in approximately 0.5 s, while individual voltage updates occur on the order of 30 ms. The current bottleneck lies in the software-device chain, and employing dedicated hardware such as FPGA-based controllers would significantly increase the attainable bandwidth.

In summary, we have demonstrated on-chip polarization management, enabling stable and tunable nonlinear signal generation through feedback system integrated with auto-compensation and auto fiber-chip alignment system. Future improvements, such as incorporating dedicated hardware and advanced control algorithms, are expected to accelerate convergence and enhance conversion efficiency, thereby advancing compact and multifunctional photonic platforms for both quantum and classical applications.

## 4 Methods

### 4.1 Simulation and design

The eigenmodes and their corresponding effective indices required to calculate the phase matching conditions shown in the PPLN and the widths of PSR were determined using COMSOL Multiphysics. All mode propagation parameters were obtained from Lumerical MODE Solver.

### 4.2 Device fabrication

The electrodes for electrical poling were patterned using PMMA resist via an e-beam lithography (EBL) system. The 100 nm thick Cr deposition followed by metal lift-off is performed, by which the Cr metal electrodes with periodic poling is formed. We perform a series of electrical pulses by applying high-voltage pulses, enabling the periodical domain inversion of the straight ridge waveguide. After poling process, the Cr electrodes are removed by using Cr etchant. The aligned EBL is required in order to create the patterns of the straight waveguides inside of the poled regions. The e-beam HSQ resist followed by Espacer conductive polymer is coated onto the TFLN wafer. After the thick HSQ resist is exposed using the EBL system, the Espacer is easily removed by DI water, and the remaining HSQ works as a mask after development of the HSQ by developer. An inductively coupled plasma-reactive ion etching (ICP-RIE) process is carried out with the remaining HSQ resist that protects the TFLN layer from Ar ions. The remaining HSQ resist is fully removed by buffer oxide etchant (BOE). The residues from TFLN waveguide are removed by KOH cleaning. The 2 μm thick upper SiO_2_ cladding layer is deposited via a plasma-enhanced chemical vapor deposition (PECVD) system. Finally, annealing process is carried out under 600 °C for an hour in N_2_ atmosphere. A chromium-based TO heater is formed directly above the top SiO_2_ cladding layer.

### 4.3 Device measurement

An experimental setup for SHG characterization is illustrated in Supporting Figure S6(a). A tunable telecom laser source (Santec TSL-550) serves as the pump. To provide adequate optical power for propagation through the TFLN waveguide, the output is amplified using an erbium-doped fiber amplifier (EDFA, Pritel LNHP-PMFA-33). A 99:1 tap coupler is positioned between the EDFA and the input lensed fiber to monitor the coupled pump power. The amplified pump light is then launched into the TFLN waveguide via a telecom lensed fiber, and the transmitted light is collected at the output using another telecom lensed fiber. The polarization state on the Poincaré sphere was measured in free space using a half-wave plate, a quarter-wave plate and a polarizer. After passing through these elements, the beam was detected by a photodetector, and the Stokes parameters were calculated using the corresponding mathematical expressions. The heater voltages and the auto fiber-chip alignment system are controlled through a DAQ devices (NI USB-6003, NI USB-6212) interfaced with Python.

## Supplementary Material

Supplementary Material Details
