# Supplementary material for: On-chip polarization management for stable nonlinear signal generation in thin-film lithium niobate
Source: Nanophotonics. 2025 Nov 10;14(26):4691–701. doi: 10.1515/nanoph-2025-0339 (PMC12714033; doi:10.1515/nanoph-2025-0339)
Supplement: Supplementary file 1 — Supplementary Material Details [file j_nanoph-2025-0339_suppl_001.pdf]

Supporting Information: On-chip Polarization Management for Stable Nonlinear Signal Generation in Thin-Film Lithium Niobate

Junhyung Lee,[1,2,*] Sunghyun Moon,[1,*] Yongchan Park,[1,2] Uijoon Park,[1,2] Hansol Kim,[1] Changhyun Kim,[1] Minho Choi,[1,3] Jin-Il Lee,[1,4] Hyeon Hwang,[5] Min-Kyo Seo,[5] Dae-Hwan Ahn,[1,6] Hojoong Jung,[1,†] and Hyounghan Kwon[1,6,‡]

[1]*Center for Quantum Technology, Korea Institute of Science and Technology (KIST), Seoul 02792, South Korea*

[2]*School of Electrical Engineering, Korea University, Seoul 02841, South Korea*

[3]*Department of Artificial Intelligence Semiconductor Engineering, Hanyang University, Seoul, 04763, South Korea*

[4]*Division of Nano Information Technology, KIST School, Korea University of Science and Technology, Seoul 02792, Republic of Korea*

[5]*Department of Physics, Korea Advanced Institute of Science and Technology (KAIST), Daejeon, 34141, South Korea*

[6]*Division of Quantum Information, KIST School, Korea University of Science and Technology, Seoul 02792, South Korea*

[*] Contributed equally

[†] Co-Corresponding author: H.J.: hojoong.jung@kist.re.kr

[‡] Co-Corresponding author: H.K.: hyounghankwon@kist.re.kr

Supporting Note 1: Design and performance of polarization splitter rotator (PSR)

Figure S1(a) shows the structure of the polarization-splitter–rotator (PSR) chip and the associated mode-conversion process. The region where TM_0 is converted to TE_1 is referred to as the rotating section, while the region where TE_1 is converted to TE_0 is referred to as the splitting section. Figures S1(b) and S1(c) plot the effective indices of the top and bottom waveguides in the rotating and splitting sections, respectively. In the figures, When the effective index curves of the two modes get closer in the plots, modal phase matching is attained, which enables efficient mode conversion between those modes. Figures S1(d) and S1(e) present the measured extinction ratios for TE_0 and TM_0 inputs. The extinction ratio for TE_0 input is approximately 20 dB, whereas for TM_0 input it is approximately 10 dB.

Although simulations predict an extinction ratio of ≈50 dB for the PSR, the experimentally measured extinction typically falls in the 10–20 dB range. This discrepancy arises primarily from practical limitations. Fabrication tolerances such as small deviations in waveguide width and gap, reduce mode-conversion efficiency and polarization purity, while fiber-based coupling and characterization add further measurement uncertainty. In particular, accurately measuring polarization extinction above ≈20 dB is difficult with conventional fiber-based setups, making it challenging to realize and verify extinction ratios beyond this range in our current experimental configuration.

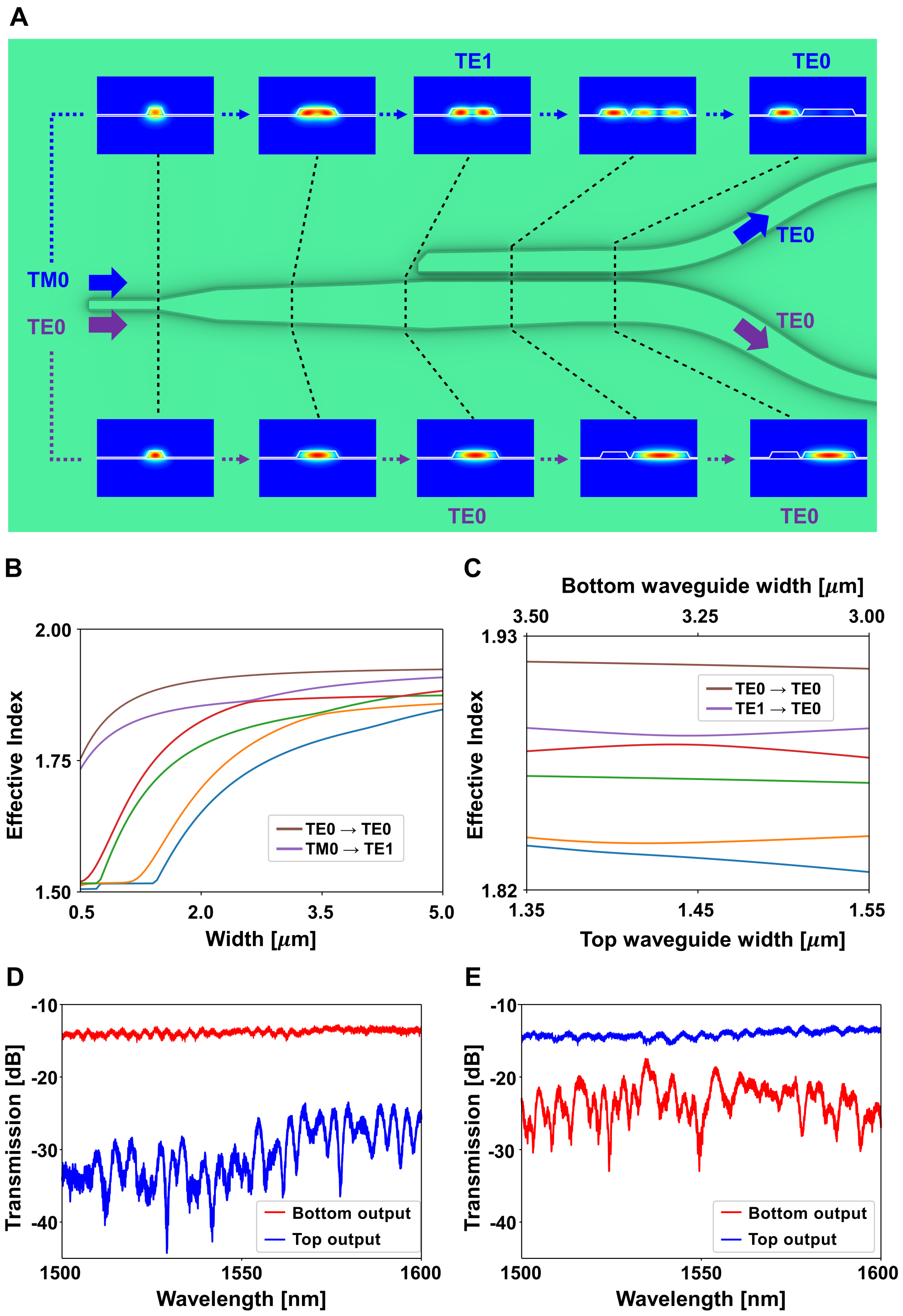

Figure S1 Design and performance of PSR. (a) Schematic of the PSR and corresponding mode propagation. **(b)** Effective refractive indices (n_{eff}) of the fundamental modes in rotating section as a function of waveguide widths. **(c)** Effective refractive indices (n_{eff}) of the fundamental modes in splitting section as a function of top and bottom waveguide widths. Extinction ratios between the top and bottom waveguides for **(d)** TE_0 and **(e)** TM_0 inputs.

Supporting Note 2: Design of PPLN

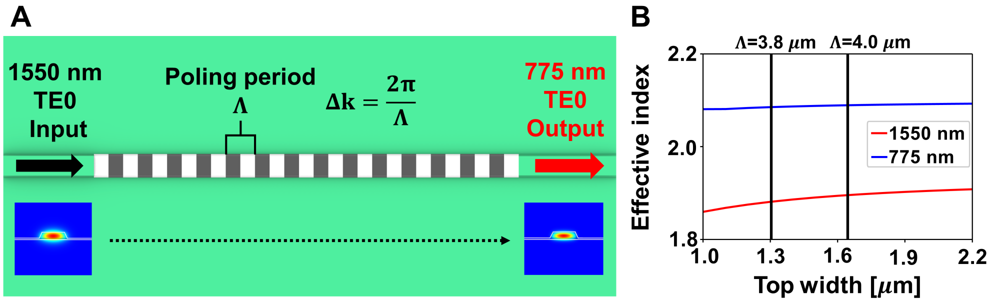

Figure S2 (a) Schematic of the type-0 PPLN waveguide for SHG. **(b)** Effective refractive indices (n_{eff}) of the fundamental modes at 1550 nm (color: red) and 775 nm (color: blue) as a function of waveguide widths.

Figure S2(a) illustrates the PPLN waveguide design used for second-harmonic generation employing the QPM technique. The structure, formed by periodically inverting the sign of the second-order nonlinear coefficient with period Λ, introduces an effective grating wavevector $2\pi/\Lambda$ that offsets the phase mismatch $\Delta k = k_{2\omega} - 2k_{\omega}$. Propagation constants obey

$$k = \frac{2\pi n_{\text{eff}}}{\lambda},$$

where n_{eff} denotes the mode effective index at each wavelength. The mode indices at 1550 nm and 775 nm are plotted versus waveguide width in Fig. S2(b). The resulting dispersion curves allow the poling period Λ required for QPM to be determined as a function of width, highlighting the flexibility of QPM-based design.

Our devices incorporate complex PSR and MZI structures, so a direct calculation of absolute SHG efficiency is difficult and the measured total loss at 1550 nm is 34 dB. To provide a reliable reference of the nonlinear conversion efficiency, we fabricated an additional PPLN waveguide under nominally identical conditions (same LN film thickness of 500 nm, same poling recipe, and same etching recipe) and measured its normalized SHG efficiency in straightforward waveguides to be approximately 790 % $\text{W}^{-1}\text{cm}^{-2}$.

Supporting Note 3: Fabrication procedures of TFLN devices and PPLN

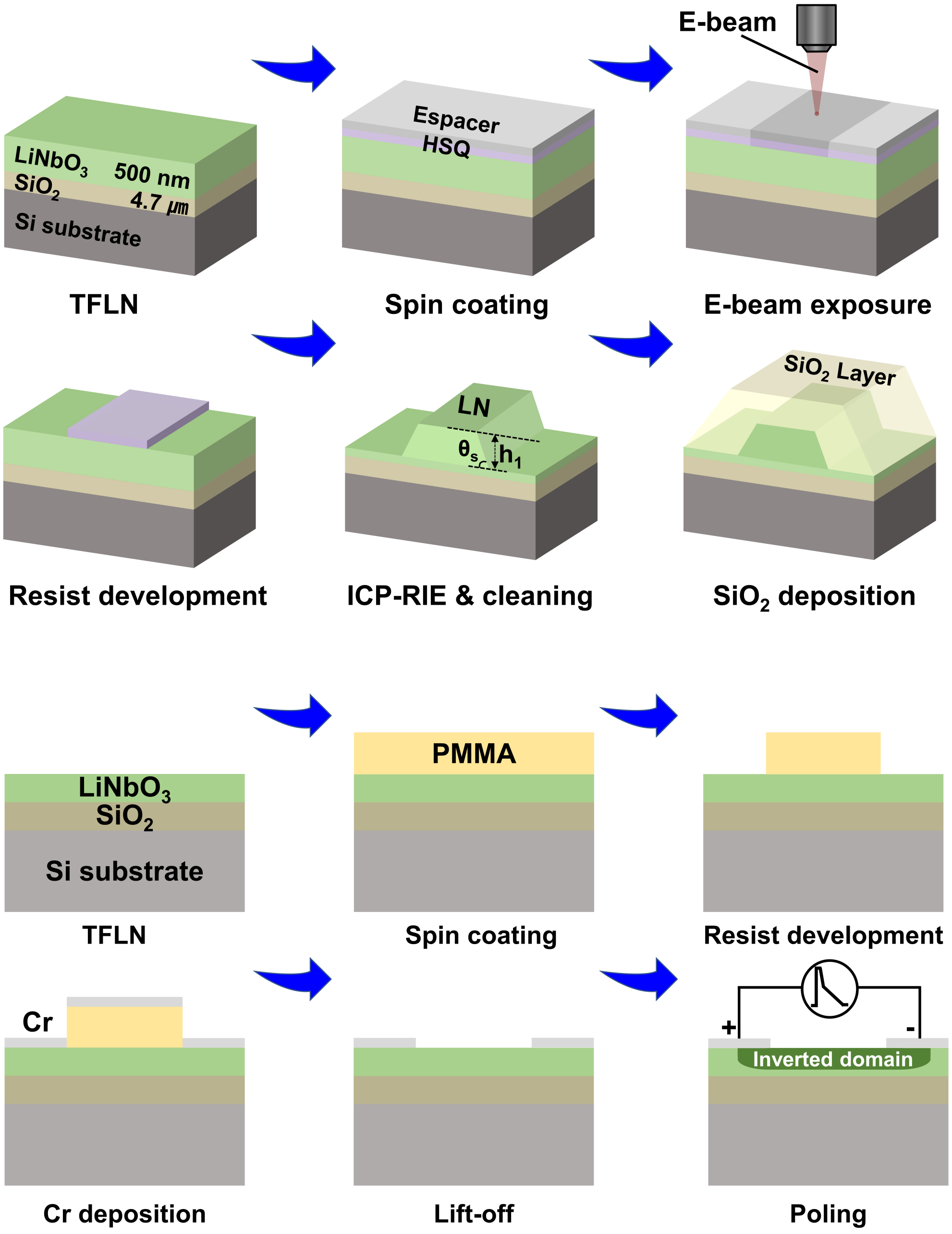

Figure S3 Fabrication procedures of TFLN devices and PPLN.

Supporting Note 4: Stokes parameters

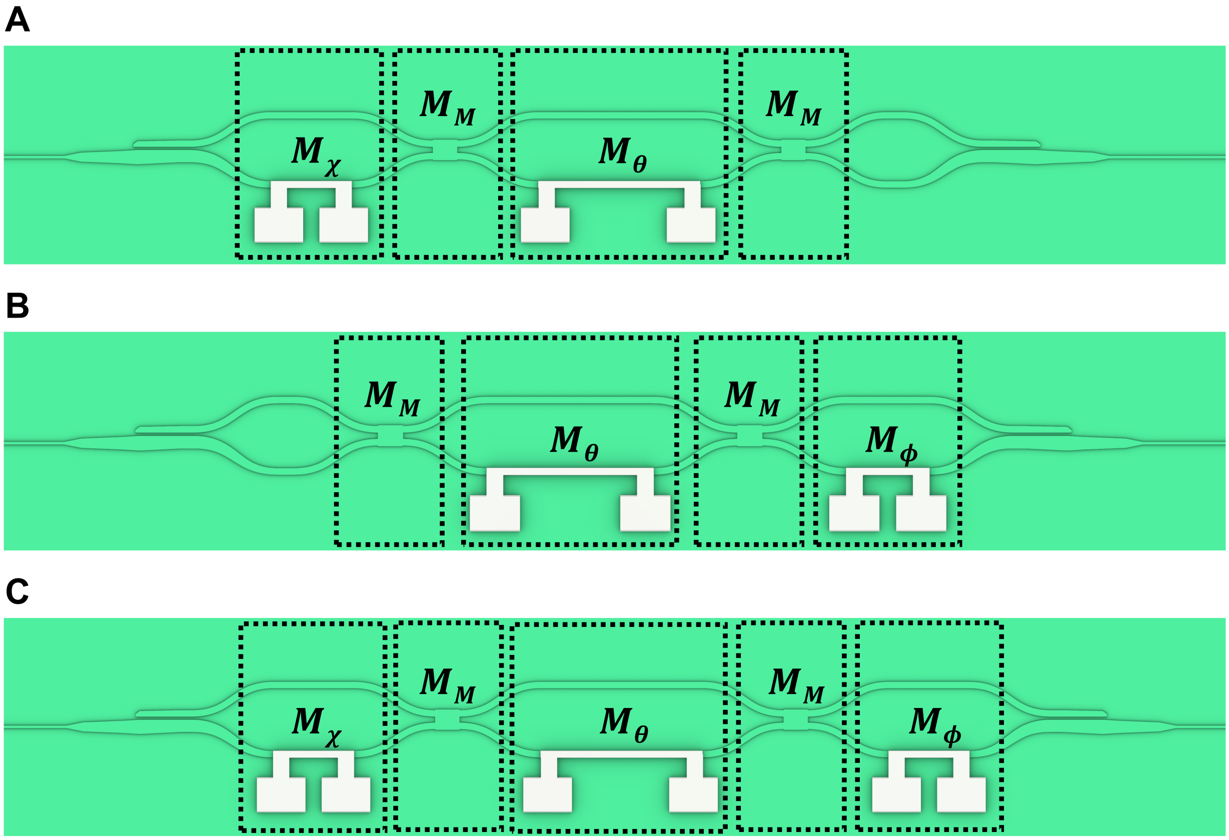

Figure S4 Polarization modulation. Three types of device configurations for polarization control: **(a)** modulation from an arbitrary input state to any linear polarization; **(b)** modulation from a linear input state to any arbitrary polarization; and **(c)** modulation from an arbitrary input state to any arbitrary polarization.

In our active polarization modulator architecture, we consider three configurations in which on-chip heaters are placed at different locations along one arm. In Figure S4(a), heaters are located at the upstream and central regions. In Figure S4(b), they occupy the central and downstream regions. In Figure S4(c), all three regions (upstream, center, and downstream) are equipped with heaters. We denote the Mueller matrices of the upstream, central, and downstream heaters by M_χ, M_θ, and M_ϕ, respectively, and the Mueller matrix of each multimode interferometer (MMI) is M_M. The input Stokes vector is given by

$$\mathbf{S}_{\text{in}} = \begin{pmatrix} S_0 \\ S_1 \\ S_2 \\ S_3 \end{pmatrix} = \begin{pmatrix} a \\ b \\ c \\ d \end{pmatrix}$$

and M_χ, M_θ, M_ϕ and M_M is defined as

$$M_\chi = \begin{pmatrix} 1 & 0 & 0 & 0 \\ 0 & 1 & 0 & 0 \\ 0 & 0 & \cos\chi & \sin\chi \\ 0 & 0 & -\sin\chi & \cos\chi \end{pmatrix}, \quad M_\phi = \begin{pmatrix} 1 & 0 & 0 & 0 \\ 0 & 1 & 0 & 0 \\ 0 & 0 & \cos\phi & \sin\phi \\ 0 & 0 & -\sin\phi & \cos\phi \end{pmatrix},$$

$$M_\theta = \begin{pmatrix} 1 & 0 & 0 & 0 \\ 0 & 1 & 0 & 0 \\ 0 & 0 & \cos\theta & \sin\theta \\ 0 & 0 & -\sin\theta & \cos\theta \end{pmatrix}, \quad M_M = \begin{pmatrix} 1 & 0 & 0 & 0 \\ 0 & 0 & 0 & -1 \\ 0 & 0 & 1 & 0 \\ 0 & 1 & 0 & 0 \end{pmatrix}.$$

Figure S4(a) places heaters before and after the first MMI and before the second MMI, yielding a total transfer matrix

$$M_{\text{total}} = M_M\, M_\theta\, M_M\, M_\chi = \begin{pmatrix} 1 & 0 & 0 & 0 \\ 0 & -\cos\theta & \sin\theta\cos\chi & \sin\theta\sin\chi \\ 0 & \sin\theta & \cos\theta\cos\chi & \cos\theta\sin\chi \\ 0 & 0 & -\sin\chi & -\cos\chi \end{pmatrix},$$

and

$$\mathbf{S}_{\text{out}} = M_{\text{total}}\,\mathbf{S}_{\text{in}} = \begin{pmatrix} S'_0 \\ S'_1 \\ S'_2 \\ S'_3 \end{pmatrix} = \begin{pmatrix} a \\ -(b\cos\theta - c\,\sin\theta\cos\chi - d\,\sin\theta\sin\chi) \\ -(-b\sin\theta - c\,\cos\theta\cos\chi - d\,\cos\theta\sin\chi) \\ -(-c\,\sin\chi + d\,\cos\chi) \end{pmatrix}.$$

For H or V inputs ($c = d = 0$), the output circular component $S'_3 = 0$, meaning circular polarization cannot be produced, therefore, Figure S4(a) represents the minimal structure capable of realizing any arbitrary linear output polarization from any arbitrary input.

Figure S4(b) instead places heaters before and after the second MMI, giving

$$M_{\text{total}} = M_\phi\, M_M\, M_\theta\, M_M = \begin{pmatrix} 1 & 0 & 0 & 0 \\ 0 & -\cos\theta & \sin\theta & 0 \\ 0 & \sin\theta\cos\phi & \cos\theta\cos\phi & -\sin\phi \\ 0 & -\sin\theta\sin\phi & -\cos\theta\sin\phi & -\cos\phi \end{pmatrix}.$$

The resulting output Stokes vector is

$$\mathbf{S}_{\text{out}} = M_{\text{total}}\,\mathbf{S}_{\text{in}} = \begin{pmatrix} S'_0 \\ S'_1 \\ S'_2 \\ S'_3 \end{pmatrix} = \begin{pmatrix} a \\ -\left(b\cos\theta - c\sin\theta\right) \\ b\,\sin\theta\cos\phi + c\,\cos\theta\cos\phi - d\,\sin\phi \\ -b\,\sin\theta\sin\phi - c\,\cos\theta\sin\phi - d\,\cos\phi \end{pmatrix}.$$

For circular inputs ($b = c = 0$), the horizontal–vertical output component $S'_1 = 0$, meaning H/V polarization cannot be produced. Figure S4(b) represents the minimal structure capable of covering the full range from linear to circular output polarization when driven with arbitrary linear input.

Figure S4(c) equips all three heater locations. The overall Mueller matrix becomes

$$\begin{aligned} M_{\text{total}} &= M_\phi\, M_M\, M_\theta\, M_M\, M_\chi \\ &= \begin{pmatrix} 1 & 0 & 0 & 0 \\ 0 & -\cos\theta & \sin\theta\cos\chi & \sin\theta\sin\chi \\ 0 & \sin\theta\cos\phi & \sin\chi\,\sin\phi + \cos\chi\,\cos\phi\,\cos\theta & \sin\chi\,\cos\phi\,\cos\theta - \sin\phi\,\cos\chi \\ 0 & -\sin\phi\,\sin\theta & \sin\chi\,\cos\phi - \sin\phi\,\cos\chi\,\cos\theta & -\sin\chi\,\sin\phi\,\cos\theta - \cos\chi\,\cos\phi \end{pmatrix}, \end{aligned}$$

giving output

$$\mathbf{S}_{\text{out}} = M_{\text{total}}\,\mathbf{S}_{\text{in}} = \begin{pmatrix} S'_0 \\ S'_1 \\ S'_2 \\ S'_3 \end{pmatrix}$$

$$= \begin{pmatrix} a \\ -\left(b\cos\theta - c\sin\theta\cos\chi - d\sin\theta\sin\chi\right) \\ b\sin\theta\cos\phi + c\left(\sin\chi\ \sin\phi + \cos\chi\ \cos\phi\ \cos\theta\right) + d\left(\sin\chi\ \cos\phi\ \cos\theta - \sin\phi\ \cos\chi\right) \\ -b\sin\phi\ \sin\theta + c\left(\sin\chi\ \cos\phi - \sin\phi\ \cos\chi\ \cos\theta\right) + d\left(-\sin\chi\ \sin\phi\ \cos\theta - \cos\chi\ \cos\phi\right) \end{pmatrix}.$$

If any two of the input-Stokes variables vanish, no single output component S'_1, S'_2, or S'_3 remains identically zero. Therefore, Figure S4(c) is a fully universal polarization synthesizer capable of mapping any arbitrary input state to any arbitrary output state.

Supporting Note 5: Extra measurement of stokes parameter

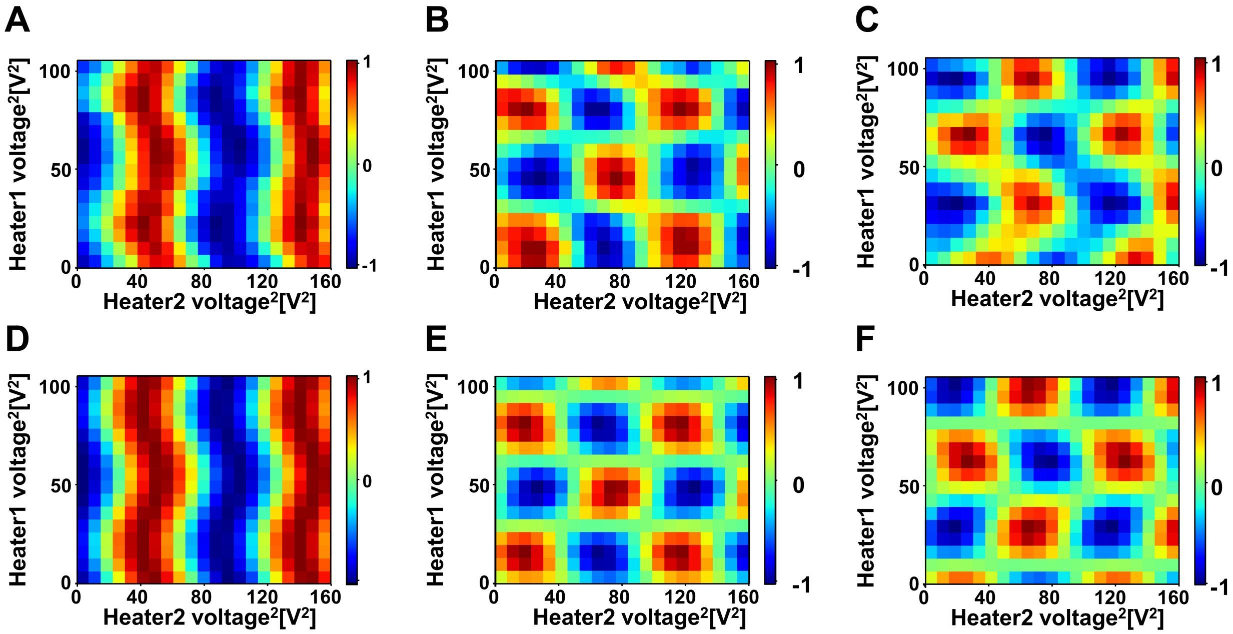

Figure S5 Measured and fitted Stokes parameters for Figure S4(b) under TE_0 input polarization. Panels **(a)–(c)** show the experimentally measured Stokes parameters **(a)** S_1, **(b)** S_2, and **(a)** S_3 as functions of the applied heater voltages. Panels **(d)–(f)** display the corresponding theoretical fits for **(d)** S_1, **(e)** S_2, and **(f)** S_3 derived from the measurement.

Supplementary Figures S5(a)–(c) present the experimentally measured Stokes parameters S_1, S_2, and S_3 as a function of the applied heater voltages under TE_0 input polarization. Figures S5(d)–(f) show the corresponding theoretical fits obtained from the measurement.

Focusing on Figure S4(b), which theoretically enables complete modulation from linear to circular polarization states, we observe a notable deviation from the expected behavior. For example, when measuring S_1 under TE_0 input polarization, where the Stokes vector reduces to $(a, b, c, d) = (1, 1, 0, 0)$, the output S_{out} is

$$\mathbf{S}_{\text{out}} = M_{\text{total}}\,\mathbf{S}_{\text{in}} = \begin{pmatrix} S'_0 \\ S'_1 \\ S'_2 \\ S'_3 \end{pmatrix} = \begin{pmatrix} 1 \\ -\cos\theta \\ \sin\theta\cos\phi \\ -\sin\theta\sin\phi \end{pmatrix}.$$

Figure S5(a) and S5(d) correspond to the normalized Stokes parameter S'_1, Figures S5(b) and S5(e)

correspond to S_2', and Figures S5(c) and S5(f) correspond to S_3'. Hence, by examining the voltage applied to Heater1 and Heater2 in each panel, one can directly infer the evolution of the output polarization state from purely linear through intermediate elliptical states to purely circular, in excellent agreement with the theoretical model.

However, closer inspection of the data reveals small but systematic deviations from this ideal behavior. Despite the expectation that S_1' should remain constant as θ varies, we observe a weak modulation of S_1' when changing ϕ, and both S_2' and S_3' exhibit Bessel-like oscillatory ripples around their theoretical curves. These discrepancies indicate that the phase shifts induced by each heater are not entirely independent. Instead, when one heater is driven, it perturbs adjacent waveguide sections owing to reflection, thereby introducing unintended phase shifts in the other arms.

Supporting Note 6: Extra measurement of SHG intensity

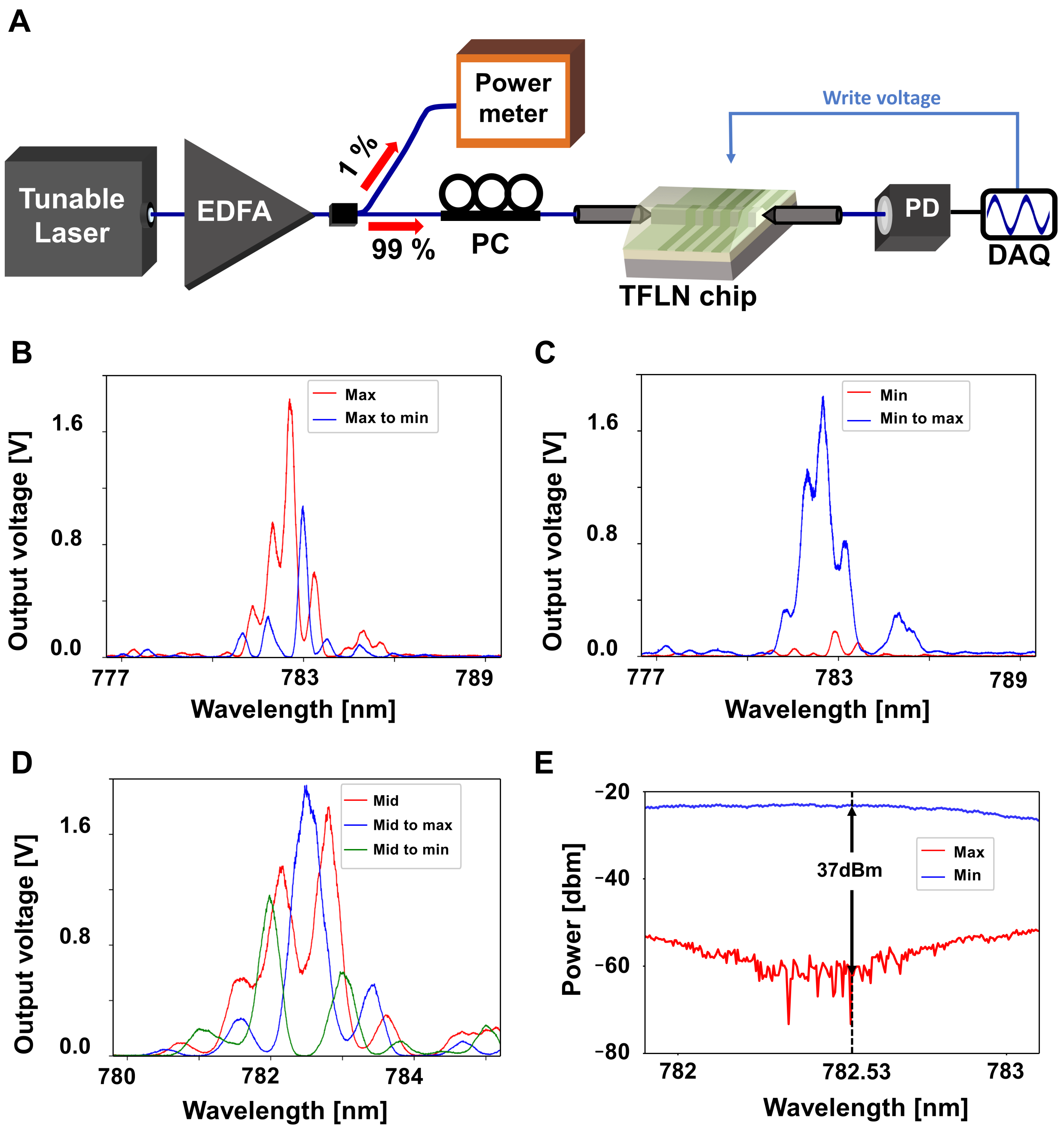

Figure S6 SHG intensity modulation (a) a schematic of the experimental setup used to measure the SHG. (b) Minimizing SHG intensity from maximum. (c) Maximizing SHG intensity from minimum. (d) Minimizing and maximizing SHG intensity from intermediate states. (e) Extinction ratios obtained from off-chip polarization controller

Figure S6(a) illustrates the measurement setup used to modulate second-harmonic generation (SHG) intensity via heater-based polarization control. Arbitrary input polarization states are

converted by the heaters into specific linear polarizations, and the resulting SHG intensity depends on the alignment between that polarization and the nonlinear crystal's phase-matching condition.

Specifically, configuration in Figure S4(a), which represents the minimal structure capable of generating arbitrary linear polarization outputs from arbitrary input states, is utilized to modulate SHG intensity between a maximum to a minimum by sweeping through linear polarization states. The figure illustrates this modulation behavior near the fundamental wavelength of 782 nm, showing how the SHG intensity varies depending on the polarization state. In all three configurations shown in Figure S6(b–d), the device exhibits polarization-state–dependent SHG control. Configuration Fig. S6(b) enables transitions between the minimum and maximum SHG intensities, whereas Fig. S6(c) and Fig. S6(d) extend the control to achieve minimization and two-way modulation from intermediate states, as discussed. Figure S6(e) shows that the extinction ratios obtained using the on-chip and off-chip polarization controllers are comparable.

Supporting Note 7: Extra measurement of SHG intensity feedback

Supplementary Figure S7(a) illustrates our automated, closed-loop feedback system for controlling intermediate transmission intensity via an on-chip phase modulator. An integrated photodetector continuously measures the transmitted signal, and a control computer adjusts the heater voltage to converge on a desired extremum, dynamically compensating for thermal drift, mechanical vibration, and other disturbances without manual intervention.

For maximization in Figure S7(b), the controller initiates at the nominal phase-matching point and takes a simple directional search. It incrementally increases the heater voltage and monitors the transmission and when the measured transmission first declines, indicating passage beyond the peak, marks this voltage as the "near-extreme" point. This point then serves as the center for the Nelder–Mead simplex optimization. Within this local region, three trial voltage vertices are evaluated at each iteration, and the simplex moves toward the vertex yielding the highest transmission while minimizing the spread of values. The time trace in Figure S7(b) demonstrates that, from the initial directional guess to final convergence, the system rapidly reaches and stabilizes at the maximum transmission.

For minimization in Figure S7(c), an analogous protocol is employed but in the opposite direction. The controller decreases the heater voltage until the transmission reading rises for the first time, identifying that voltage as the near-extreme trough. The Nelder–Mead algorithm is then launched around this point, contracting the simplex toward successively lower transmission readings and reduced variance. Panel Figure S7(c) shows the resulting suppression of transmission, with the system holding the low-intensity state stably over time.

In both routines, feedback computations run on a control computer using custom Python scripts interfaced to a high-bandwidth DAQ module and a DC power supply to drive the heater. However, the response time of the DC power supply that applies voltage to the heater currently limits the overall feedback-loop speed, constraining real-time optimization performance. By improving the dynamic response of the heater power source, faster and more stable feedback control can be achieved.

Figures S7(d) and (e) show the simultaneous variations of the SHG and telecom pump intensities during the feedback process. Because coupling and transmission losses are polarization-dependent, polarization changes also affect pump transmission, yielding extinction ratios of 5.0 dB under increasing conditions and 3.5 dB under decreasing conditions. The asymmetry between these

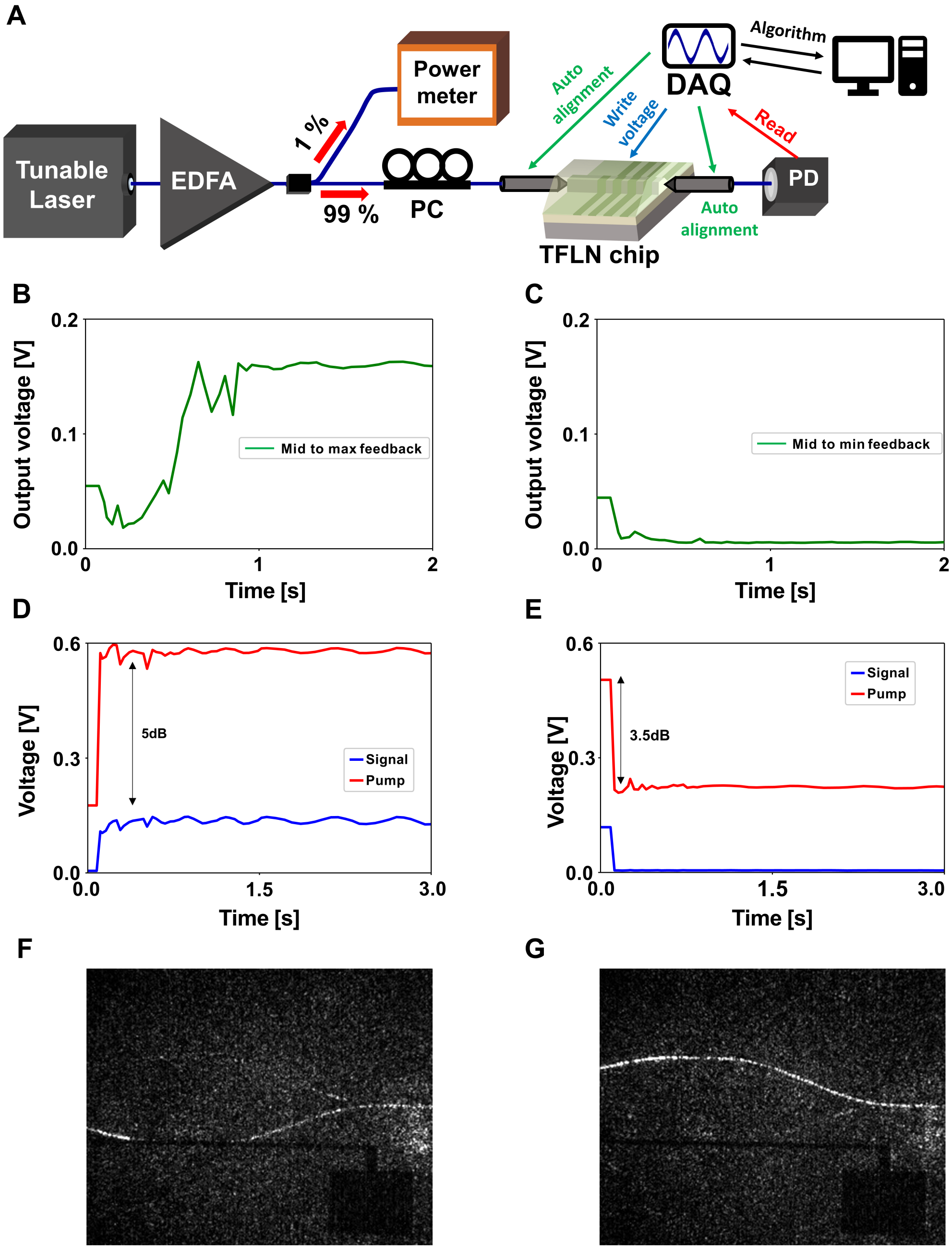

Figure S7 Automated feedback modulation of SHG intensity from an intermediate operating point. **(a)** A schematic of the experimental setup for measuring SHG intensity using a feedback system integrated with auto-alignment and auto-compensation. **(b)** Transition from the mid-level SHG signal to its maximized intensity under closed-loop control. **(c)** Transition from the mid-level SHG signal to its minimized intensity under closed-loop control. **(d)** Transition of pump and signal intensities from the minimum SHG level to the maximized intensity under closed-loop control. **(e)** Transition of pump and signal intensities from the maximum SHG level to the minimized intensity under closed-loop control. **(f)** Optical microscopy image when SHG optimized for maximum output. **(g)** Optical microscopy image when SHG optimized for minimum output.

values reflects differences in coupling and propagation losses, so the SHG extinction cannot be

explained merely as twice the pump extinction. The much larger SHG extinction observed in Figs. 3(b) and (c), compared with the pump extinction and the full Stokes-parameter measurement shown in Fig.2, confirms that the dominant contribution to the observed SHG modulation arises from polarization control rather than incidental pump-power fluctuations. Telecom-band camera images recorded at the SHG-optimized maximum and minimum in Figs. S7(f) and (g) further support that the SHG signal is governed primarily by polarization control rather than by transmission variations.
